# Parametric excitation and squeezing in a many-body spinor condensate

**DOI:** 10.1038/ncomms11233

**Published:** 2016-04-05

**Authors:** T. M. Hoang, M. Anquez, B. A. Robbins, X. Y. Yang, B. J. Land, C. D. Hamley, M. S. Chapman

**Affiliations:** 1 School of Physics, Georgia Institute of Technology, Atlanta, Georgia 30332-0430, USA

## Abstract

Atomic spins are usually manipulated using radio frequency or microwave fields to excite Rabi oscillations between different spin states. These are single-particle quantum control techniques that perform ideally with individual particles or non-interacting ensembles. In many-body systems, inter-particle interactions are unavoidable; however, interactions can be used to realize new control schemes unique to interacting systems. Here we demonstrate a many-body control scheme to coherently excite and control the quantum spin states of an atomic Bose gas that realizes parametric excitation of many-body collective spin states by time varying the relative strength of the Zeeman and spin-dependent collisional interaction energies at multiples of the natural frequency of the system. Although parametric excitation of a classical system is ineffective from the ground state, we show that in our experiment, parametric excitation from the quantum ground state leads to the generation of quantum squeezed states.

## Abstract (web summary)

Ultracold atomic gases with collisional interactions allow the exploration of quantum many-body physics. Here, the authors vary over time the contributions to the interaction energy to coherently control the spin state of an atomic Bose gas, thereby implementing a truly many-body control scheme.

## Graphical abstract

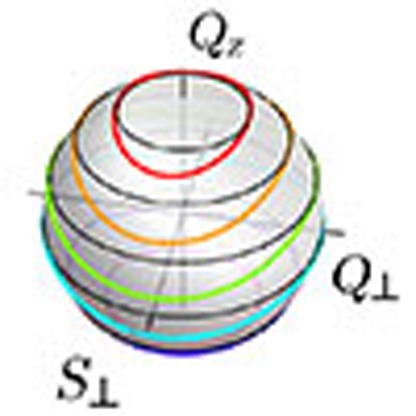

Parametric excitation of an oscillating physical system can be achieved by periodically varying one of its parameters to modulate the natural frequency of the oscillator, *f*_0_; a textbook example is a simple pendulum excited by modulating its length, ℓ, such that 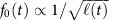 (ref. 1). A fundamental distinction between parametric excitation and direct excitation by periodic forcing is shown in Fig. 1, which shows instantaneous phase space orbits of a simple oscillator for the two cases. For direct excitation, the applied force periodically displaces the equilibrium position of the oscillator, leaving the orbits otherwise unchanged, and efficient excitation occurs when the excitation frequency matches the natural frequency of the oscillator, *f*=*f*_0_. For parametric excitation, the parameter modulation leaves the equilibrium location unchanged but instead periodically distorts the phase orbits; in this case, efficient excitation occurs for excitation frequencies *f*=2*f*_0_/*n*, *n*=1,2,3…

Ultracold atomic gases with well-characterized collisional interactions allow new explorations of non-equilibrium dynamics of quantum many-body physics and for synthesis of strongly correlated quantum states including spin-squeezed^2^,^3^,^4^,^5^ and non-Gaussian entangled states^6^,^7^,^8^ relevant for quantum sensing^9^ and quantum information^10^. In ultracold atom traps, parametric excitation of the atomic motion, achieved by modulating the trapping potential, is used to measure the trap frequency as well as in a variety of studies including the excitation of Bose–Einstein condensate collective density modes^11^,^12^,^13^,^14^, controlling the superfluid/Mott insulator transition^15^,^16^ and photon-assisted tunnelling in modulated optical lattices and super-lattices^17^,^18^,^19^,^20^,^21^,^22^.

In this work, we demonstrate parametric excitation of the internal states of a collection of atomic spins. The spins are coherently excited to non-equilibrium states by a simple modulation of the magnetic field magnitude at very low frequencies (<200 Hz) compared with the energy difference of the Zeeman states (Δ*E*/*h*=0.7 MHz, where *h* is Planck's constant). The excitation spectrum is fully characterized and compares well to theoretical calculations. Parametric excitation of the ground state is also investigated. Classically, parametric modulation of an oscillating system does not excite the ground state^1^. Here, we show that the finite quantum fluctuations of the collective spin leads to parametric excitation of the ground state, which manifest as exponential evolution of the fluctuations and the generation of non-classical squeezed states. The exponential evolution and squeezing of the spin fluctuations are measured and agree qualitatively with theory. Finally, we discuss how these techniques can be applied to related systems including the double-well Bose–Hubbard model and interacting (psuedo) spin-1/2 ensembles.

## Results

### Experimental system

**Figure 1:**
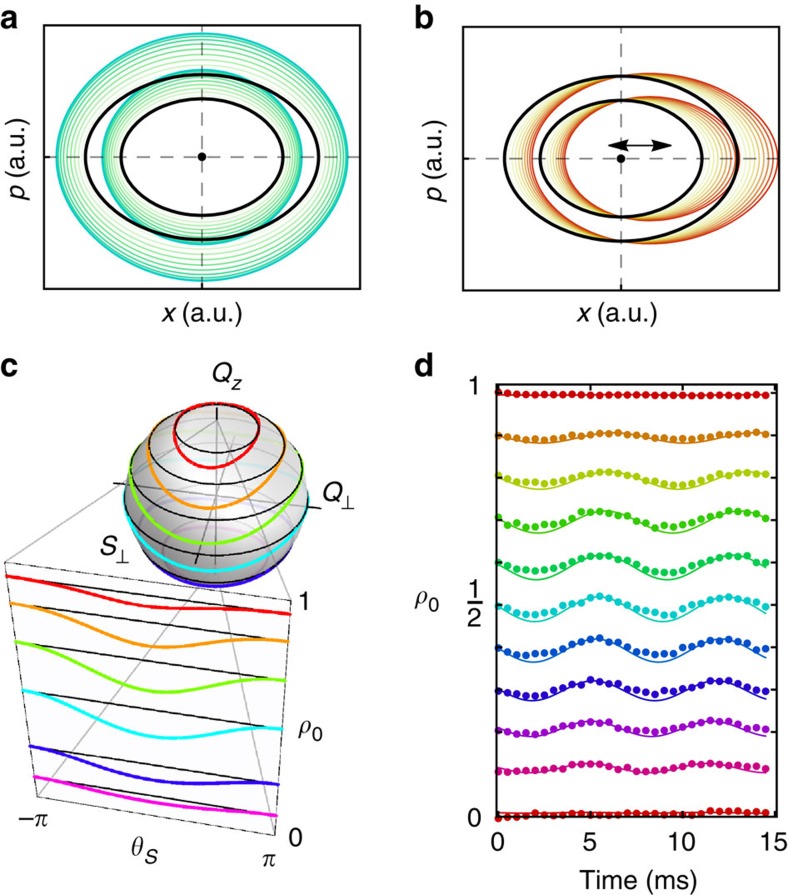
Experimental concept. Momentum-position phase space of a harmonic oscillator under (**a**) parametric and (**b**) direct excitation. The original orbits are shown in black, and the modified orbits are shown in colour at different instants of the periodic excitation. (**c**) The phase space of the condensate. The collective states (normalized to *N*) lie on a unit sphere with axes *S*_⊥_, *Q*_⊥_, *Q*_*z*_. The orbits of constant energy for non-interacting (*c*=0) spins are lines of latitude shown in black and the orbits for interacting spins with *q*=10|*c*| are shown in colour. The *ρ*_0_, *θ*_*s*_ diagram is a Mercator projection of the hemisphere. *θ*_*s*_=*θ*_+1_+*θ*_−1_−2*θ*_0_ is the relative phase of the three Zeeman sub-levels, *m*_*F*_=0,±1. (**d**) Measurements of the natural oscillations of *ρ*_0_ at *B*=1 G for different initial states *ρ*_0_(*t*=0)∈[0, 1]. The experimental data (markers) are compared with simulations (lines). Unless otherwise indicated, the uncertainty in the measurement of *ρ*_0_ is <1%.

The experiments use ^87^Rb Bose condensates with *N*=40,000 atoms in the *F*=1 hyperfine level tightly confined in optical traps such that spin domain formation is energetically suppressed and dynamical evolution of the system occurs only in the internal spin variables. The Hamiltonian describing the evolution of this collective spin system in a bias magnetic field *B* along the *z*-axis is^5^,^23^,^24^,^25^:


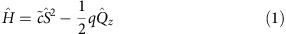


where 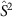 is the total spin-1 operator and 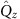 is proportional to the spin-1 quadrupole moment, 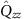. The coefficient 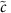 is the collisional spin interaction energy per particle integrated over the condensate and *q*=*q*_*z*_*B*^2^ is the quadratic Zeeman energy per particle with *q*_*z*_=72 Hz G^−2^ (hereafter, *h*=1). The longitudinal magnetization 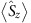 is a constant of the motion (=0 for these experiments); hence, the first-order linear Zeeman energy 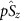 with *p*∝*B* can be ignored. The spin-1 coherent states can be represented on the surface of a unit sphere shown in Fig. 1(c) with axes {*S*_⊥_,*Q*_⊥_,*Q*_*z*_}, where *S*_⊥_ is the transverse spin, 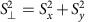, *Q*_⊥_ is the transverse off-diagonal nematic moment, 
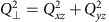
, and *Q*_*z*_=2*ρ*_0_–1, where *ρ*_0_ is the fractional population in the *F*=1, *m*_*F*_=0 state. In this representation, the dynamical orbits are the constant energy contours of 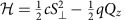, where 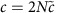.

The experiment is conducted at high fields where the Zeeman energy dominates the spin interaction energy, *q*/|*c*|=10. In this regime, the lowest energy state is the polar state (*ρ*_0_=1) located at the top of the sphere, and the dynamical orbits of the excited states to leading order are simple rotations about the *Q*_*z*_ axis with a frequency *f*_0_≈*q*+*cQ*_*z*_ (see the Methods for details). Despite the small relative magnitude of the spin interaction term, it has the important effect of breaking the polar symmetry and thereby slightly distorting the orbits from the latitudinal lines of the sphere. As the state orbits the sphere, the population *ρ*_0_ undergoes small periodic nutations at twice the orbit frequency, as shown in the *ρ*_0_, *θ*_*s*_ projection in Fig. 1(c). The maximum nutation amplitude is Δ*ρ*_0_≈0.02 for *ρ*_0_=0.5 and goes to zero for *ρ*_0_=0,1. Measurements of these distortions are shown in Fig. 1(d) for different initial values of *ρ*_0_.

### Parametric excitation

**Figure 2:**
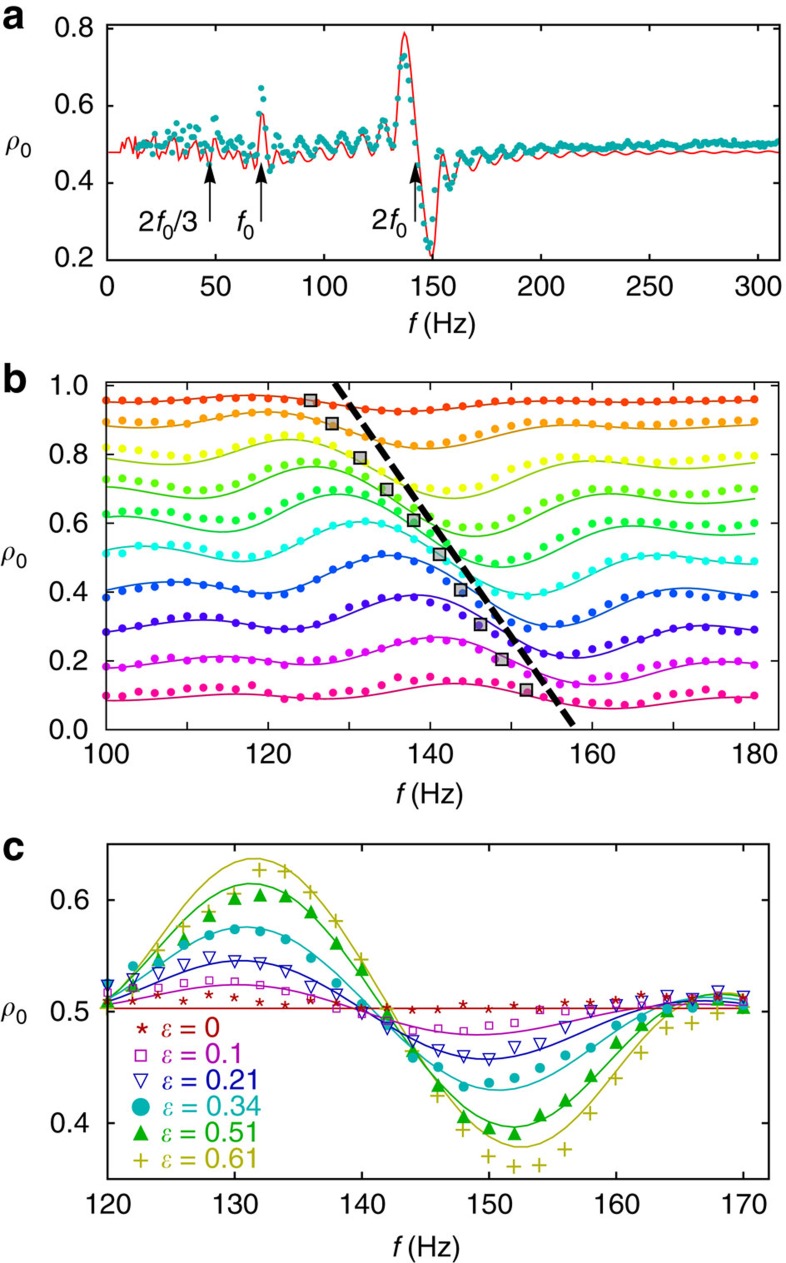
Demonstration of parametric excitation. (**a**) Population *ρ*_0_ after 100 ms of parametric excitation for (*ρ*_0_(0), *φ*_0_, *ɛ*)=(0.5, *π*, *ɛ*=0.5). Data (markers) are plotted with simulation (solid line) for comparison. The excitation spectrum shows clear resonances at frequencies 2*f*_0_ and *f*_0_. (**b**) Populations *ρ*_0_ after 40 ms of parametric excitation for different initial *ρ*_0_ populations for *φ*_0_=*π* and *ɛ*=0.5. Data (markers) are compared with simulation (solid lines), and hue colours correspond to the initial *ρ*_0_∈[0, 1]. The dashed line is the theoretical prediction for 2*f*_0_ resonance, and the square boxes indicate the location of the measured resonance. (**c**) Population *ρ*_0_ after 40 ms of parametric excitation for different modulation amplitudes *ɛ*.

Parametric excitation requires periodic modulation of one of the parameters of the Hamiltonian; in a spin-1 condensate described by equation (1), this is conveniently achieved by modulating the bias magnetic field and hence the quadratic Zeeman energy term, *q*(*t*)∝*B*^2^(*t*). The condensate is first prepared in a coherent state with *ρ*_0_, *θ*_*s*_=(0.5, *π*) at a field of 1 G, corresponding to an initial quadratic Zeeman energy *q*_0_=72 Hz. The spinor dynamical rate is *c*=−7(1) Hz, determined from measurements of coherent oscillations at low fields. To parametrically excite the spins, the magnetic field is modulated for a duration of time, after which the spin populations are measured to determine the final value of *ρ*_0_. The applied modulation is harmonic in *q* and has the form *q*(*t*)=*q*_0_[1+*ɛ*sin (2*πft*–*φ*_0_)]. The measured excitation spectrum versus modulation frequency is shown in Fig. 2(a). The spectrum shows the characteristic features of parametric excitation, namely strong excitation at 2*f*_0_=142 Hz and weaker excitation at *f*_0_. Other resonances are theoretically observable at smaller *f*=2*f*_0_/*n* values; however, they are dominated by the tails of the more prominent peaks making them difficult to detect. The experimental data (marker) are compared with a simulation using equation (1) (solid line) and show good agreement overall.

Beyond comparing the experimental results to numerical solutions of the quantum Hamiltonian, insight into the parametric excitation is obtained by considering the mean-field dynamical equations for *ρ*_0_ and the quadrature angle *θ*=*θ*_s_/2 (ref. 26):


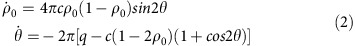


These equations are similar to bosonic Josephson junction equations describing the double-well condensate^27^,^28^ and can be solved like-wise by integrating the phase and using the Jacobi-Anger expansion (see the Methods for details),


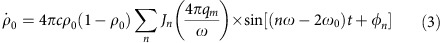


where *J*_*n*_ is the Bessel function of order *n*, *q*_*m*_=*ɛq*_0_ is the modulation strength and *φ*_*n*_ depends on the initial conditions including the phase of the modulation (see the Methods for details). The parametric resonance frequencies are obvious from this solution because the time-average of 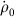 is zero unless 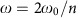.

In the 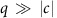 high field regime of these experiments, the collisional interactions shift the natural oscillation frequency as 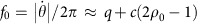 to lowest order. This shift is investigated in Fig. 2(b), where the excitation is measured for different initial values of *ρ*_0_. The hue colours correspond to the initial *ρ*_0_ values using the same scale as the coherent oscillation data in Fig. 1(d), and the square markers indicate the positions of the measured resonance frequencies where Δ*ρ*_0_≈0. The measured resonance frequencies are in good overall agreement with the expected dependence on the initial value of *ρ*_0_ shown with the dashed line—the small discrepancy is attributed to an inductive delay in the excitation of ∼1 ms (see the Methods for details) that creates a phase offset 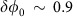 rad in the excitation. Indeed, the experimental data compare very well to simulations (solid line) that include this phase offset.

The dependence of the excitation amplitude on the drive strength *q*_*m*_=*ɛq*_0_ is reflected in the Bessel function 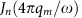. In Fig. 2(c), the excitation is measured for different modulation amplitudes. The experimental data (markers) are compared with the simulations (solid line) and show good agreement (see the Methods for details). As expected, the modulation amplitude does not affect the resonance frequency of the parametric excitation; however, increasing the modulation amplitude results in larger excitation of *ρ*_0_.

**Figure 3:**
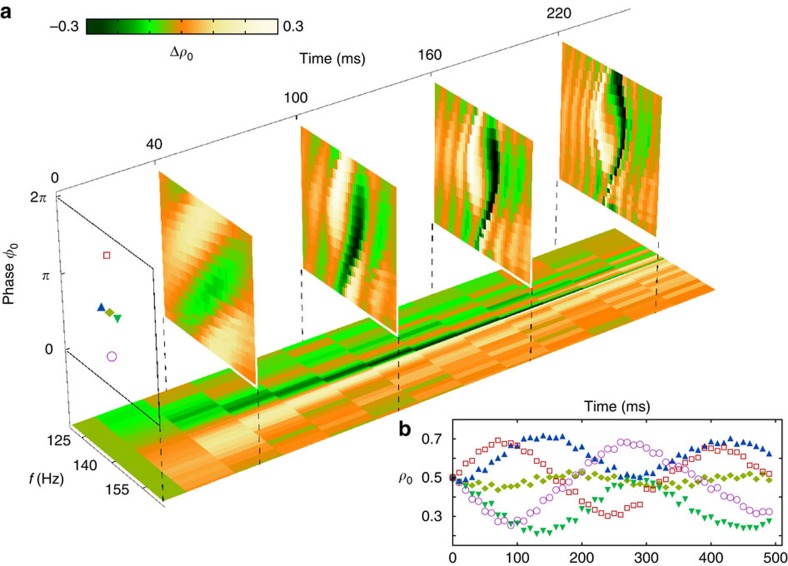
Excitation parameter map. (**a**) The data show the excitation of the condensate, Δ*ρ*_0_=*ρ*_0_−*ρ*_0_(0) following parametric excitation as a function of the excitation frequency *f* and initial phase *φ*_0_ of the modulation. The initial state is *ρ*_0_(0),*θ*_*s*_=(0.5,*π*) and the modulation strength is *ɛ*=0.5. The four vertical slices show Δ*ρ*_0_ after 40, 100, 160 and 220 ms of parametric excitation. The horizontal plot shows the evolution of Δ*ρ*_0_ for an initial phase *φ*_0_=0. (**b**) The temporal evolution of *ρ*_0_ for different *f*, *φ*_0_ pairs indicated in the vertical slice in **a**. The markers on the inset correspond to the markers on the main diagram.

Parametric excitation is a coherent process, and hence it can be employed as a tool for quantum control of the collective spin. However, accurate control requires detailed knowledge of the system response to the excitation parameters. In Fig. 3b, we present a parameter variation map that shows the excitation in the neighbourhood of the 2*f*_0_ resonance for different values of excitation time *t*, phase *φ*_0_ and frequency *f*. For each measurement, we prepare the initial state *ρ*_0_, *θ*_s_=(0.5, *π*) using an radio frequency (rf) pulse and modulate the quadratic Zeeman energy at different frequencies and initial phases. The change in population Δ*ρ*_0_ is measured after excitation times of 40, 100, 160 and 220 ms as shown in Fig. 3a (four vertical slices). The white/orange (black/green) regions represent the positive (negative) changes in population. These two regions evolve and spiral to form a distinctive ‘yin-yang' pattern. This pattern in the measurements agrees well with theoretical calculations of the excitation (see the Methods for details). At the centre of the pattern (*f*, *φ*_0_)=(2*f*_0_, *π*) where 2*f*_0_≈143 Hz, *ρ*_0_ remains unchanged and the excitation shows anti-symmetry about this point (diamond marker). Figure 3b shows the temporal evolution of *ρ*_0_ for different *f*, *φ*_0_ pairs indicated in the vertical slice. Although in each case the initial state is identical, the final state after excitation shows a strong dependence on both the frequency and phase of the applied modulation. In addition, we show a map of the population dynamics for the initial phase *φ*_0_=0 (Fig. 3a, horizontal slide). The two distinguishable domains, white (orange) and black (green), are separated by the resonance frequency. The population dynamics exhibit oscillations during the excitation process, and, approaching the resonance frequency, both the oscillating period and the amplitude (Δ*ρ*_0_) increase.

### Generation of squeezed states

We now turn to measurements of uniquely quantum features of the excitation. For a classical oscillator prepared in its stable equilibrium configuration, an important distinction between direct excitation and parametric excitation is that former can efficiently excite the oscillator while the latter cannot. The equilibrium (ground) state is a stable fixed point in phase space and if the oscillator is perfectly initialized, it will remain unexcited by parametric modulation. However, for a quantum system prepared in its ground state, intrinsic Heisenberg-limited fluctuations of the state still allow for parametric excitation. In the semi-classical picture, the quantum fluctuations populate a family of orbits about the equilibrium point in the phase space that can be parametrically excited.

**Figure 4:**
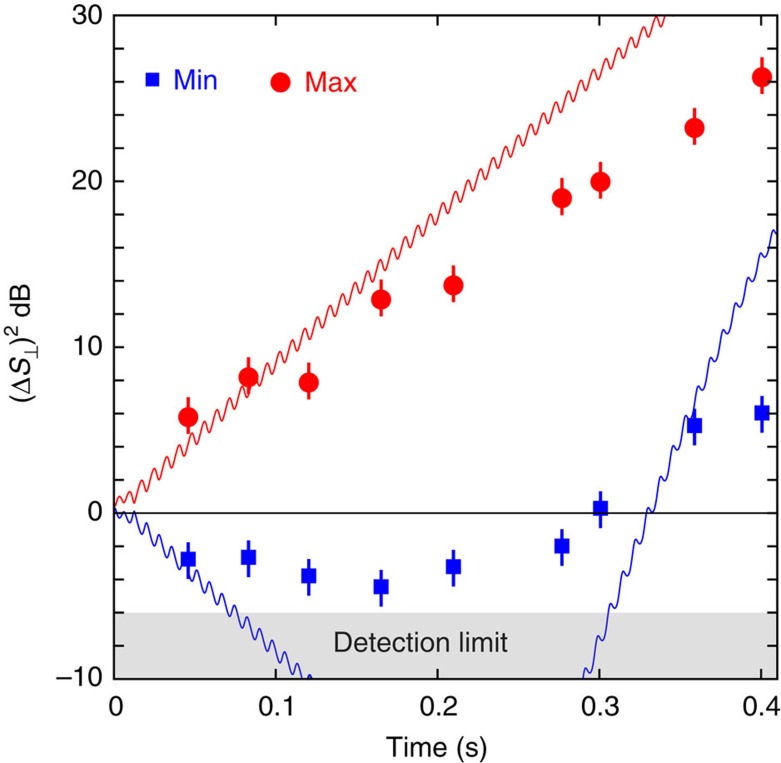
Parametric excitation from *ρ*_0_=1. Evolution of the minimum and maximum values of the transverse spin fluctuations, 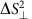, following parametric excitation with *ɛ*=0.56 and *f*=134 Hz starting from the initial state *ρ*_0_=1. The measured maximum (Max; red markers) and minimum (Min; blue markers) variance of the transverse magnetization *S*_⊥_ are compared with simulation (solid red and solid blue). The error bars indicate the s.e. of the measured variances determined from 30 repeated measurements per data point.

In Fig. 4, we investigate parametric excitation from the quantum ground state of the condensate located at *ρ*_0_=1 and demonstrate that parametric excitation can be used to generate quadrature squeezed states. Although the population, *ρ*_0_, is largely insensitive to parametric excitation from the *ρ*_0_=1 state (in contrast with the *ρ*_0_≠1 initial states), the fluctuations in the transverse coordinates, *S*_⊥_, *Q*_⊥_, evolve exponentially with time and show quadrature squeezing in the spin-nematic phase space. In contrast to our previous demonstration of squeezing^5^, in which the squeezing was generated by free dynamical evolution following a quench that localized the state at a unstable (hyperbolic) fixed point, here, the squeezing is generated near a stable fixed point by periodic distortion of the phase space orbits produced by the modulation of the quadratic Zeeman energy. The essential difference is the time dependence in the Hamiltonian.

In Fig. 4, measurements of the minimum and maximum values of the quadrature fluctuations of the transverse spin, Δ*S*_⊥_, are shown and compared with a quantum simulation. As evidenced by both the measurements and the calculations, the fluctuations in the initial state evolve exponentially at early evolution times and develop into quadrature squeezed states. The maximum squeezing measured is −5 dB, which is close to the detection-limited ceiling of −6 dB because of the photo-detection shot-noise and background scattered light^5^. The simulations suggest that with technical improvements, the system is capable of generating squeezing at the −20 dB level. Although the experimental data show the main effects predicted by theory, the agreement of the measured fluctuations with the theory is not perfect, particularly at longer evolution times. This is possibly due to effects of atom loss from the condensate, which has a lifetime of 1.5 s for these experiments, and we plan to further investigate this question in the future work.

In summary, we have demonstrated a mechanism for control and excitation of an ensemble of spins based on parametric excitation. This is a many-body control technique that relies on spin-dependent collisional interactions, which we have characterized for a wide range of control parameters. We have shown that this method, when applied to the ground state, can be used to generate squeezed states.

## Discussion

The parametric excitation can also be understood as transitions between eigentates of the many-body Hamiltonian, which can be calculated by diagonalizing the tridiagonal matrix^6^,^29^


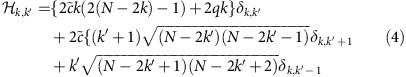


written in the Fock basis |*N*, *M*; *k*〉, where *k* is the number of pairs of *m*_*f*_=±1 atoms, *N* is the total number of atoms and *M* is the magnetization; both *N* and *M* are conserved by the Hamiltonian. Treating 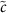 as a perturbation, the eigenenergies are 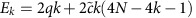 and the energy difference between Fock states is ∂*E*_*k*_/∂*k*=2*q*+2*c*(2*ρ*_0_−1) (see the Methods for details). Using this picture, we note that the parametric excitation spectrum excitation frequencies *f*=2*f*_0_/*n*, *n*=1,2,3… corresponds to many photon excitations of the system with *f*=2*f*_0_ being the single photon transition.

It is interesting to contrast parametric excitation with the usual Rabi excitation of 2-level atomic spins using rf or microwave magnetic fields. In parametric excitation, the time variation of a parameter modifies the Hamiltonian without displacing the equilibrium (ground) state of the system. As we have demonstrated that this can be achieved in a spin-1 condensate by simply modulating the magnitude of the bias magnetic field, which modulates the quadratic Zeeman energy term in the Hamiltonian. As the field strength is varied, the ground state remains at the pole of the sphere, while the shape of the orbits is modulated; this is similar to the case shown in Fig. 1(a). Rabi excitation of 2-level atomic spins using rf or microwave magnetic fields, on the other hand, is direct excitation rather than parametric excitation. Although in both cases the excitation employs time-varying magnetic fields, in the Rabi case, the oscillating magnetic field is transverse to the bias field, which leads to a oscillation of the orientation of the total field. Because the ground state of the spin aligns along the field direction, the addition of the time-varying transverse field leads to a periodic displacement of the ground state away from the pole in the usual Bloch sphere picture of the spin vector, similar to the case shown in Fig. 1(b).

We point out that the techniques we have demonstrated are applicable to related many-body systems including the double-well Bose-Hubbard (or Bosonic Josephon junction model), collisionally interacting pseudo-spin 1/2 two component condensates and ensembles of spin-1/2 atoms with photon-mediated interactions. Each of these systems can be described by a version of the Lipkin–Meshkov–Glick model Hamiltonian^30^, 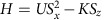, whose the mean-field phase space is functionally identical to the spin-nematic phase space shown in Fig. 1(c)^5^,^28^. At their heart, these systems feature competing energy terms (one of which is non-linear) that give rise to a quantum critical point. The *q*>2|*c*| polar phase that we explore in this work corresponds to the Rabi regime (*K*>*U*) in the Bosonic Josephon junction system, which is the tunnelling-dominated regime perturbed by the interactions 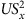, and the modulation of *q* corresponds to a modulation of the tunnelling coefficient *K*. Indeed, there have been numerous theoretical proposals for excitations of these systems using periodic modulations (for example, see refs 31, 32, 33, 34 for details), and many of the lattice-based experimental demonstrations mentioned previously realize closely related ideas generalized to multi-site systems^15^,^16^,^17^,^18^,^19^,^20^,^21^,^22^.

## Methods

### Experimental concept

The experiment utilizes ^87^Rb atomic Bose–Einstein condensates created in an optical trap containing *N*=40,000 atoms initialized in the |*F*=1, *m*_*F*_=0〉 hyperfine state in a high magnetic field (2 G). To prepare the initial spin state, the condensate is rapidly quenched to a magnetic field of 1 G, and a Rabi rf pulse resonant with the *F*=1 Zeeman transition is applied to prepare the desired initial state *ρ*_0_, *θ*_*s*_=(*ρ*_0_(*t*=0), *π*). The nominal value of the magnetic field *B*=1 G is determined by using rf and microwave spectroscopy, and the spinor dynamical rate is determined by measuring coherent spin dynamics oscillations using states prepared near the ferromagnetic ground state (*c*=−7(1) Hz).

Parametric excitation of the system is implemented by sinusoidally modulating *q*, which is implemented by time-varying the magnetic field using external coils. Owing to induced eddy currents in the metal vacuum chamber and the inductance of the magnetic field coils, the applied modulation is time-delayed relative to the intended control by 1 ms and reduced in amplitude by 15%. These effects are measured directly using magnetically sensitive rf spectroscopy of the atoms, and they are incorporated in all the simulations.

The final spin populations of the condensate are measured by releasing the trap and allowing the atoms to expand in a Stern–Gerlach magnetic field gradient to separate the *m*_*F*_ spin components. The atoms are probed for 400 μs with three pairs of counter-propagating orthogonal laser beams, and the fluorescence signal is collected by a CCD camera is used to determine the number of atoms in each spin component.

To measure the transverse spin fluctuations Δ*S*_⊥_, an rf Rabi *π*/2 pulse is applied during the expansion to rotate the transverse spin fluctuations (which are in the *x*,*y* plane) into the *z* measurement basis. The fluctuations are then determined from 30 repeated measurements of 〈*S*_*z*_〉, the difference in the number of atoms measured in the *m*_*F*_=1 and *m*_*F*_=−1 spin components.

### Coherent oscillation dynamics

We first discuss parametric excitation using semi-classical mean field theory. The excitation occurs when the quadratic Zeeman energy is modulated at integer divisors of twice the natural coherent oscillation frequency in the (*θ*, *ρ*_0_) phase space. The dynamics of the system are governed by a set of differential equations for the fractional population *ρ*_0_ and the phase *θ* from refs 35, 36


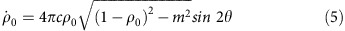



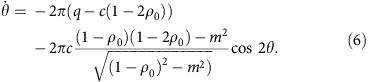


where 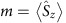 and we have taken *h*→1. The spinor energy of the system is given by


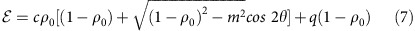


and has an oscillation period^35^ of the form


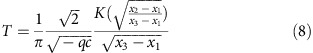


where *K*(*k*) is the elliptic integral of the first kind and *x*_*i*_ are the roots of the differential equation


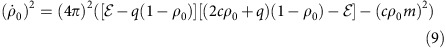


For a condensate prepared in *m*_*F*_=0, with magnetization conserved *m*=0, as is the case in our experiment, the roots *x*_*i*_ are


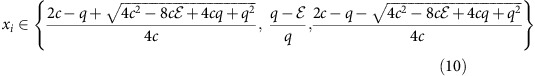


In order to calculate the period *T*, we first calculate


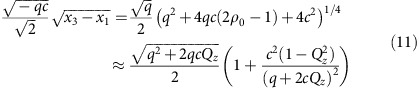


where *Q*_*z*_=2*ρ*_0_−1. The elliptical integral part of the period in equation (8)


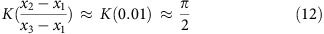


Substituting these results back into the equation for the period, we obtain the natural coherent oscillation frequency in quadrature phase (*θ*, *ρ*_0_)


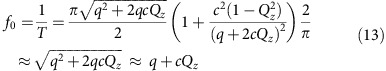


where we have that 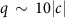 in our experiment.

Parametric excitation of the system is achieved by periodically modulating the quadratic Zeeman term *q* in the Hamiltonian. Efficient excitation occurs when the modulation frequency is an integer divisor of twice the natural frequency of the system 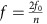, where 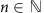. In our system, the coherent oscillations occur at a magnetic field *B*=1 G and spinor dynamical rate *c*=−7.2(5) Hz. These parameters yield a range of natural frequencies


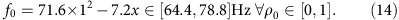


with the most dominant excitation frequency corresponding to *n*=1


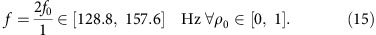


### Parametric excitation theory

In our experiment, the system is prepared in the *m*_*F*_=0 state with magnetization *m*=0 and allowed to evolve at sufficiently high fields, where *q*/|*c*|=10. Under these conditions, equation (5) simplifies to


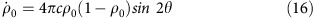



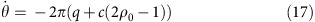


Parametric excitation is then applied by modulating the quadratic Zeeman energy


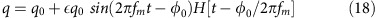


The Heaviside function and phase *φ*_0_ imply that the modulation starts at *q*_0_ and is active for 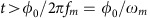, where 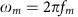. We prepare an initial *ρ*_0_ at high field using an rf pulse, which initializes the quadrature phase *θ*_0_=*π*/2. The system then freely evolves for 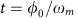, advancing the quadrature phase 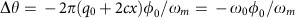, followed by modulation of *q*, where we have implicitly assumed that for evolution at high field we can set *ρ*_0_ to be a constant so that integration of 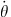 is straightforward. We therefore have two initial contributions to the quadrature phase before modulation, namely *θ*_0_ and Δ*θ*.

For evolution times 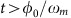, we can integrate 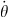 in equation (16) giving:


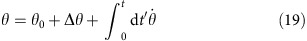



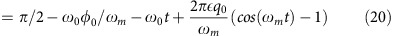


where 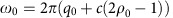. Substituting the phase into the population dynamical equation (16), we obtain


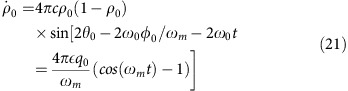



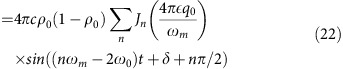


where the phase 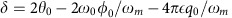. In equation (22), we have made use of the identity sin(*A*+*B*)=sin(*A*)cos(*B*)+cos(*A*)sin(*B*) along with the Jacobi–Anger expansions


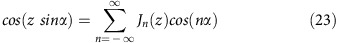



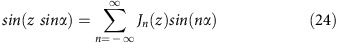


Analysing equation (22) gives us some insight into the population dynamics as a function of the modulation parameters. When 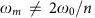, the time average of 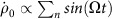 is zero. When 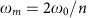, the time average of the *n*th term in the expansion 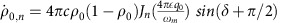 is non-zero. Therefore, only for the case when 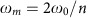 is there sufficient coupling from the modulation to parametrically excite the system. The behaviour of the Bessel functions 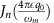 also indicates that the strongest coupling occurs for *n*=1 or 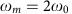, a signature of parametric excitation.

The strength of the excitation is controlled by tuning *ɛ*. When the system is modulated at 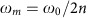 we can focus on short-time dynamics 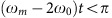, as the higher order terms are negligible becuase of time averaging, and expand equation (22) about *ɛ*=0. It can be shown that the coefficient in the expansion is exact up to 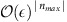. Using this fact, the population dynamical equation for 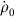 can be rewritten as:


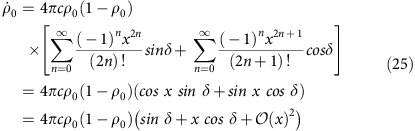


where 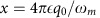. Depending on the value of 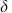, increasing the strength of the modulation *ɛ* will either enhance or reduce the response of the system to the modulation for short times. Furthermore, it is also apparent from equation (25), along with the expression for 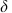, that the response of the system is periodic with respect to the initial phase of the modulation 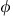, and has fixed points for *ρ*_0_=0.5 and *δ*=*nπ,* where *n* is an integer, both of which agree with the experimental data shown in Fig. 3 of the main paper.

We now consider the strong coupling case where *n*=1 or 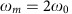, and the higher order terms |*n*|>1 of 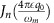 are negligible, so that


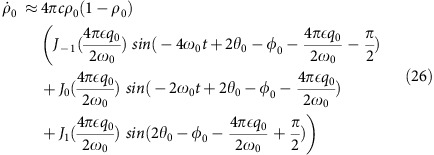


and after integration


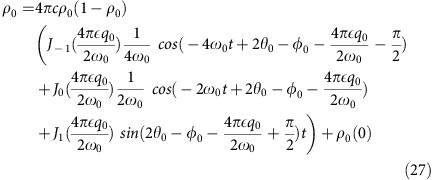


The population *ρ*_0_(*t*)≈*ρ*_0_(0) if *φ*_0_=1.37*π* and 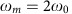.

### Quantum parametric excitation

An alternative way to view the parametric excitation is by considering transitions between the eigenstates of the many-body Hamiltonian. The energy corresponding to the oscillation frequency matches no single atom transition. Rather, it approximately matches the energy difference between two atoms in the *m*_*F*_=0 state and a pair of atoms in the *m*_*F*_=±1 states. Yet, even this is not precise enough as this energy separation also depends on the collective state of the system varying from 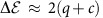 for all atoms in the *m*_*F*_=0 state to 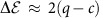 for all atoms in the *m*_*F*_=±1 states. These energy separations can be calculated by diagonalizing the tridiagonal matrix given by


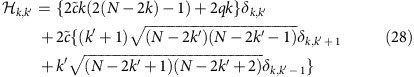


where 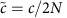 and *k* is the number of pairs of *m*_*F*_=±1 atoms in the enumeration of the Fock basis. The Fock basis, |*N*, *M*, *k*〉, is also enumerated with *N* the total number of atoms, and *M* the magnetization, both of which are conserved by the Hamiltonian leaving all dynamics in *k*. The off-diagonal contributions in equation (28) are due to the many-body interaction given by the 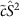 term of the Hamiltonian. This interaction results in mixing of the Fock states, even in the high field limit. Without this interaction, there would be no transitions as the magnetic interactions, both linear and quadratic Zeeman, are diagonal in the Fock basis. In this picture, the integer divisor frequencies of the spectrum correspond to many photon excitations of the system.

In the regime where *q*=*q*_*Z*_*B*^2^>2*c*, we treat 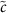 as a perturbation and 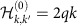 with expansion coefficients


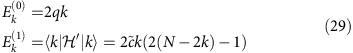


and eigenenergy of the system


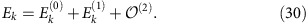


The resonance frequency between Fock states is the energy difference between each Fock state


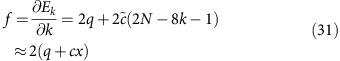


where the last line corresponds to *k*<<*N*. To first order, the resonance frequency between Fock states is the same as the frequency obtained from mean field theory equation (13). The factor of two arises from the definition of the resonant frequency *f*=2*f*_0_.

### Parameter variation map simulation

To compare the experimental data shown in Fig. 3 of the main paper to theory, we perform four semi-classical simulations at fixed evolution times that demonstrate excitation in the neighbourhood of the 2*f*_0_ resonance for different values of the modulation phase *φ*_0_ and modulation frequency *f*. For each simulation, we initialize the state (*ρ*_0_, *θ*_*s*_)=(0.5, *π*) and modulate the quadratic Zeeman energy *q* at different frequencies and phases. Details of the simulation method can be found in refs 5, 6.

**Figure 5:**
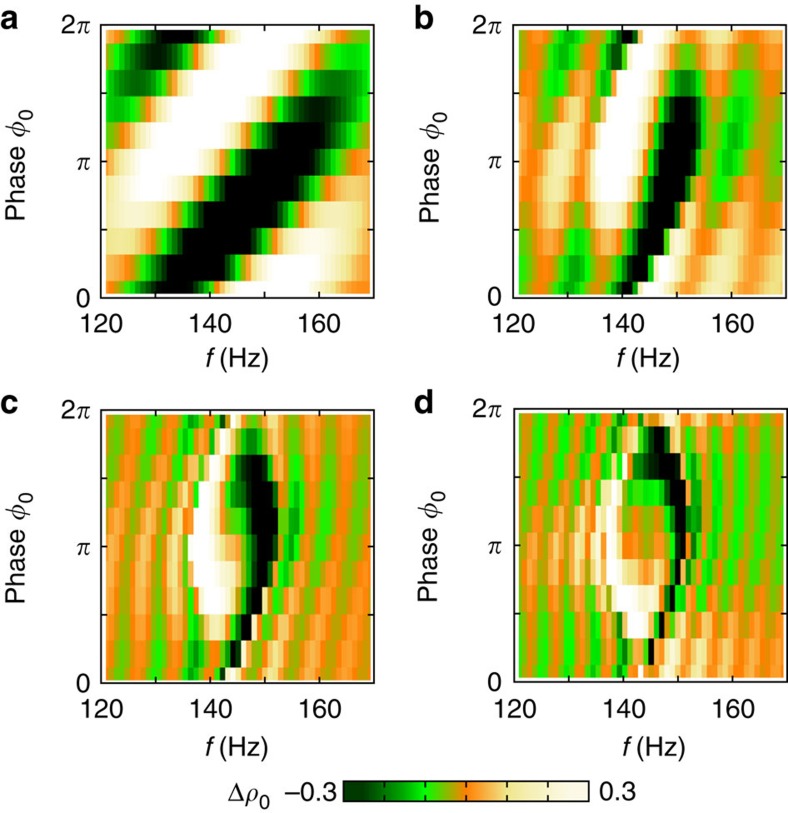
Excitation parameter map simulation. Semi-classical simulations demonstrating parametric excitation for varying modulation phase *φ*_0_ and modulation frequency *f* corresponding to evolution times of 40 ms (**a**), 100 ms (**b**), 160 ms (**c**) and 220 ms (**d**) running clockwise.

The change in population Δ*ρ*_0_ is calculated after excitation times of 40, 100, 160 and 220 ms, as shown running clockwise in Fig. 5. These simulations correspond to the vertical slices shown in Fig. 3 of the main paper, and agree quite favourably. The white/orange (black/green) regions represent the positive (negative) changes in population, which evolve and spiral to form a distinctive ‘yin-yang' pattern. At the centre of the plots (*f*, *φ*_0_)=(2*f*_0_, *π*), where 2*f*_0_≈143 Hz, *ρ*_0_ remains unchanged and the excitation shows anti-symmetry about this point.

## Additional information

**How to cite this article:** Hoang, T. M. *et al.* Parametric excitation and squeezing in a many-body spinor condensate. *Nat. Commun.* 7:11233 doi: 10.1038/ncomms11233 (2016).
